# Determinants of hospital inefficiency. The case of Polish county hospitals

**DOI:** 10.1371/journal.pone.0256267

**Published:** 2021-08-17

**Authors:** Agata Sielskas

**Affiliations:** Department of Applied Economics, Collegium of Finance and Management, Warsaw School of Economics SGH, Warsaw, Poland; University of Bologna, ITALY

## Abstract

Local hospitals play a crucial role in the healthcare system. In this study, the efficiency of Polish county hospitals is assessed by considering characteristics of hospitals that may determine their performance, such as the form of ownership, size, and staff structure. The main goal was to analyze the effect of three possible determinants on efficiency: ownership, the presence of an Emergency Department, and the presence of an Intensive Care Unit. The study covered different subgroups of hospitals and different approaches of inputs and outputs. An input-oriented radial super-efficiency DEA model under variable returns to scale was used for the efficiency analysis, and then differences between distributions of efficient and inefficient units were evaluated using a Chi-square test. A Kruskal-Wallis test was also used to analyze differences in mean efficiency. Inefficiency scores were regressed with hospital characteristics to test for other determinants. These results did not confirm differences in efficiency concerning ownership. However, in some subgroups of hospitals, running an Emergency Department or an Intensive Care Unit had a significant effect. Tobit regression results provided additional insight into how an Emergency Department or Intensive Care Unit can affect efficiency. Both cases had an effect of increasing inefficiency, and the data suggested that the department/unit size plays an important role.

## Introduction

Hospitals perform one of the most crucial roles in the healthcare system as they are often responsible for the correct diagnosis and treatment of the population. However, in Poland, this is still an area that has not yet been thoroughly analyzed. The present paper focuses on hospitals that belong to Polish counties (powiats, i.e. the second-level unit of local government and administration in Poland). Although the majority of Polish county hospitals are public units, some are commercial companies (hereafter referred to as commercialized hospitals). Between 2018 and 2019 Polish county hospitals started their own program of financial self-diagnosis. Empirical studies have shown that the form of ownership (public or commercialized) and the presence of an Emergency Department (ED) in the hospital structure has an impact on their results and costs [1].

The hospital system in Poland has undergone significant change since the introduction of national health insurance in 1997. From 1999 the payer and the supplier were different units, but in 2003 a new reform was introduced as a consequence of controversies caused by the previous change and excess regionalization. This reform introduced the Narodowy Fundusz Zdrowia (National Health Fund, hereafter abbreviated as NHF) as a single centralized payer [2], and then in 2017, the hospital network was introduced. These changes in the legal environment have been affecting how hospitals are being managed, since they have needed to adjust to new rules.

The objective of the present paper is to study the effect of three hospital characteristics on efficiency: (1) ownership (legal form: whether the hospital is public or commercialized), (2) presence of an Emergency Department, and (3) presence of an Intensive Care Unit (ICU). The hypothesis is that all of these factors will have an impact on efficiency.

The efficiency determinants discussed in the international literature include hospital ownership [3–8], case-mix index [9], teaching status [5,8,10–12], hospital size [5,10,11,13], staff structure [10,13], length of stay [13,14], and location [5,9,11], among others. The efficiency of Polish hospitals has been analyzed by Sielska and Nojszewska [15], while other analyses have been performed on hospital departments [16,17]. Miszczyńska [18] discussed the effectiveness of the functioning of hospitals using a multi-criteria approach, while Łagowski [19] and Ćwiąkała-Małys et al. [20] focused on selected departments. Moreover, many of the studies are focused during periods when hospitals were performing under different rules to the current ones (e.g. the study by Rój was published in 2003 [21], Łagowski focuses on the period before 2015 [19], Grzesiak and Wyrozębka on 2013 [17], and Lachowska [22] on 2009–2010). Some of the papers on Polish hospital efficiency analyzed the performance at the regional level—in these cases provinces (voivodeships, i.e. Poland’s first-level administrative regions) were compared instead of individual units [23–25]. Miszczyńska [18] is one of the latest studies to provide results that can be generalized for the whole population of Polish hospitals. Therefore, the present paper is a significant contribution towards the discussion about determinants of Polish hospitals in the literature.

Efficiency is an important concept in economics, rooted in the scarcity of resources [26]. It is defined as the ratio of outputs to inputs (1).

$$efficiency=\frac{\sum_{r=1}^{s}y_{r}}{\sum_{i=1}^{m}x_{i}}$$ (1)

where

r=1,…,s

i=1,…,m

*s*–number of outputs,

*m*–number of inputs,

*x*_*i*_–value of i-th input,

*y*_*r*_–value of r-th output

The literature on hospital efficiency analysis provides several examples of relevant inputs and outputs. Outputs include variables reflecting inpatient and outpatient treatment (measured in days, visits or number of patients), the number of different medical procedures, and variables that describe the use of beds, such as the occupancy rate or average length of stay. Factors such as the numbers of beds and employees (primarily doctors and nurses) are usually used as inputs [27–29].

An increase in efficiency may result from either a decrease of inputs or an increase of outputs; or both simultaneously. For a hospital, it is difficult to set inputs at an optimal level due to the nondeterministic nature of the environment. It is usually not possible to forecast the demand for healthcare services at an individual level, and as a result, hospitals should always be prepared to provide more services than usual. However, this can lead to an excess of resources—measured both in terms of equipment as well as staff. Due to this uncertainty, both (seemingly excessive) diagnostic and treatment potentialities should be maintained or, alternatively, patients could be transported to a specialist hospital to receive adequate treatment without delay. From the efficiency standpoint, such a situation is difficult to assess because future demand is unknown. Furthermore, although the situation may seem efficient at the level of the whole system, it may be assessed as ineffective at the individual hospital level. Some local authorities may therefore find it necessary to keep the hospitals they own ready to be able to fulfil their duties without delays (compare [30,31]).

The form of ownership contributes towards the capabilities of a hospital. The hypothesis is that this will have an impact on efficiency for two reasons. Firstly, as hospitals are complex, and often highly specialized units, they are managed by appointed managers (who can either be doctors themselves or personnel who cooperate with doctors employed in the facility) instead of by the owner. This situation is known in economics as the principal-agent problem and leads to the question of whose objective function is being maximized [32] Different agents face different incentives, which may result in inefficient performance. Secondly, due to the importance of county hospitals to its local population, it can be assumed that a so-called soft budget constraint [33–35] exists within the group owned by local authorities.

It seems that the pursuit of securing the needs of the local population and providing stable employment in their region are among the most important reasons for supporting even an unprofitable hospital. A theoretical model for a soft-budget constraint in public hospitals was presented by Wright [35]. However, the empirical evidence for the effect of ownership on hospital efficiency is mixed [7]. Tiemann and Schreyögg [7] showed that the efficiency of hospitals in Germany was higher if they were publicly owned. On the other hand, Staat [6] did not find any significant effect from the type of ownership, while Kalhor et al. [4] do identify a significant impact. De Souza et al. [3] also concluded that there is different efficiency in the financial management of Brazilian hospitals concerning ownership (between voluntary and public hospitals). Valdmanis et al. [8] presented results showing that for-profit hospitals are relatively less inefficient than non-profit or public ones. Another study found ownership not to have a significant impact, although the inefficiency was higher in government-owned hospitals [5], which is aligned with the first hypothesis of the present paper.

Cheng et al. [13] provided the result that government subsidies have a negative relationship with technical efficiency, which is also interesting considering this hypothesis. In the case of Polish healthcare providers, Lachowska [22] stated that efficiency was higher in cases of non-public providers than public ones. The study, however, covered only one of the regions of Poland, West Pomeranian province. It should be noted that Łagowski [19] analyzed the efficiency of public and private hospitals operating in Dolnośląskie province, finding out that there were no differences in efficiency between these two groups. However, no statistical tests were performed.

The first hypothesis in the present paper is as follows:

H1 (ownership): efficiency of public hospitals is lower than private (commercialized, non-public) ones.

As mentioned previously, Polish counties seek to provide the most needed services and the widest possible security at the local level, which leads to the hospitals running an Emergency Department (ED) or Intensive Care Unit (ICU). Both of these units need to be kept in a state of readiness in case of emergencies, which translates to higher costs (both equipment and staff duty hours). The hypothesis is that either of these units in the structure of a hospital will lead to lower efficiency:

H2 (Emergency Department): efficiency of hospitals with an ED is lower.
H3 (Intensive Care Unit): efficiency of hospitals with an ICU is lower.

Three potential determinants studied in this paper (form of ownership, ED and ICU) were analyzed, controlling for other variables such as hospital size [5,10,11,13] and staff structure [10,13].

This paper is organized as follows; The first section presents the methodology and data. In the second section, the results are presented. The third section is dedicated to the discussion, which is then followed by conclusions.

## Materials and methods

### Data Envelopment Analysis (DEA)

DEA (Data Envelopment Analysis) was used analyze hospital efficiency. This is a nonparametric method that allows technical efficiency to be studied for multiple inputs and outputs. Along with Stochastic Frontier Analysis (SFA) [36,37], DEA is one of the most popular methods of efficiency analysis. Ravaghi et al. [28] presented a systematic review of DEA applications in the studies of hospital efficiency. In contrast to SFA, DEA does not require any assumptions about the functional form or estimation, so it can be used with smaller datasets. For this reason, DEA was chosen to be used for the current study. Efficiency was analyzed using an input-oriented radial super-efficiency DEA model, first proposed by Andersen and Petersen [38], under variable returns to scale. A super-efficiency model was chosen because it allows for differentiation between efficient units, in addition to the identification of efficient and inefficient units, which is enabled by standard DEA models [39].

DEA analysis is done in R 4.0.2 [40] using deaR package [41].

### Study design

Hospitals were divided into 3 groups using the following criteria, according to the list of possible determinants previously proposed:

- *Ownership*: public or private (non-public, performing as a commercial company). The division was based on the dummy variable COMMER, which equals 1 for commercialized hospitals and 0 otherwise.
- *Emergency Department (ED)*: with or without an ED in the structure. The division was based on the dummy variable ED, which equals 1 for hospitals with an ED and 0 otherwise.
- *Intensive Care Unit (ICU)*: with or without an ICU in the structure. The division was based on the dummy variable ICU which equals 1 for hospitals with an ICU and 0 otherwise.

The efficiency of hospitals is usually measured using inputs representing labor and capital, both in monetary and real terms, and reviews of different sets of inputs and outputs have been presented [27–29].

The present analysis was conducted using three separate approaches: (1) *general* (2) *detailed* and (3) *traditional*.

#### The *general* approach

In the first approach (hereafter referred to as *general*), efficiency was determined for the whole group of hospitals. The impact of each potential determinant on the efficiency was then studied based on the comparison between mean and median efficiency, and distributions of the shares between efficient and inefficient hospitals. The set of variables used was based on the literature (see [27–29]) with the addition of more specific inputs referring to some of the cost categories e.g. services bought by hospitals in the local market. The employment of non-health staff was also divided into separate groups. In total, one output and thirteen inputs are used. *Patient-days total* was used as the output, while the following inputs were considered: *Total Number of Beds*, *Contract with NHF*, *Materials (medical)*, *Materials (non-medical)*, *Energy*, *Outsourcing (medical)*, *Outsourcing (non-medical)*, *Doctors*, *Nurses*, *Service Workers*, *Administration Staff*, *Fixed Assets*, and *Current Assets*.

#### The *detailed* approach

The second approach (hereafter referred to as *detailed*) was based on the assumption that the sets of inputs differ between each subgroup of hospitals, and, therefore, variables should be proposed for each case individually. The efficiency was analyzed in all three groups separately, which allowed separate assessment of the impact of each determinant while controlling for other factors. Inputs and potential determinants for each subgroup of hospitals are presented in Table 1.

**Table 1:** Inputs and potential determinants by subgroup of hospitals.

Analyzed subgroup of hospitals	Potential determinants	Inputs
Public hospitals	Emergency Department	As in *general* approach
	Intensive Care Unit	
Hospitals with no Emergency Department	Ownership	As in *general* approach
	Intensive Care Unit	
Hospitals with an Intensive Care Unit	Ownership	*Non-ICU beds*, *ICU beds*, *Contract with NHF*, *Materials (medical)*, *Materials (non-medical)*, *Energy*, *Outsourcing (medical)*, *Outsourcing (non-medical)*, *Doctors*, *Nurses*, *Service Workers*, *Administration Staff*, *Fixed Assets*, *Current Assets*
	Emergency Department	

The selection of hospitals included in the analysis was conducted for each subgroup in the following way:

Step 1. Select hospitals that belong to the subgroup based on the values of dummy variable (ED, ICU, COMMER).
Step 2. Exclude hospitals for the case in which the output or any of the inputs (from the respective set of inputs) is non-positive or missing.
Step 3. Check the number of hospitals in each subgroup—if the number is lower than five in any year, discard the group; otherwise, conduct DEA analysis.

#### The *traditional* approach

In the third approach (hereafter referred to as *traditional*), the number of inputs and outputs was reduced. The goal was to enable the analysis of smaller groups of hospitals (commercialized ones, hospitals without ICU) according to the rules presented in [42]. The model used in this step was built using the inputs and outputs that often occur in the literature, that is *Nurses*, *Doctors* and the *Number of Beds* as inputs, and the number of *Patient-days Total* as a single output. The inputs in this approach were the smallest set used in similar studies (compare [27–29]).

### Tests and regression models

The impact of potential determinants on DEA score can be analyzed based on either non-parametric statistical tests [43,44] or regression models [5,9,10,13,14].

In the present analysis, the differences in mean efficiency were tested using a Kruskall-Wallis (hereafter abbreviated as KW) test. Additionally, a Pearson’s Chi-squared test (with Yates’ continuity correction if needed) was used to check whether the numbers of inefficient (i.e. efficiency below 1) and efficient (i.e. efficiency greater than or equal to 1) hospitals differ among groups.

In the final step, a Tobit model of hospital inefficiency was estimated for a whole sample of Polish county hospitals. The analysis was done with the following approach:

Step 1. Exclude hospitals for cases in which the output or any of the inputs (from the respective set of inputs) is non-positive or missing.
Step 2. Calculate DEA efficiency score for remaining Polish county hospitals (ED, ICU, COMMER).
Step 3. Exclude hospitals for cases in which DEA model is infeasible.
Step 4. Merge the DEA results with the dataset containing explanatory variables.
Step 5. Remove hospitals for cases in which any explanatory variables are missing.
Step 6. Estimate Tobit models for remaining hospitals.

This approach was designed to prevent the situation where a hospitals efficiency score could be biased due to the prior removal of a hospital due to missing explanatory variables.

The three potential determinants studied in this paper were COMMER (form of ownership), ED, and ICU. These were analyzed while controlling for other variables such as hospital size [5,10,11,13] and staff structure [10,13], which have been used as determinants in other studies.

In [5,10,13], the inefficiency score was defined as:

$$inefficiency=\frac{1}{DEA\;efficiency\;score}-1$$ (2)

Due to the superefficiency model used, in order to keep the left limit at 0, the inefficiency score was defined as:

$$inefficiency=\left\{ \begin{array}{cc} 0 & ;\;if\;DEA\;efficiency\;score\geq 1\\ \frac{1}{DEA\;efficiency\;score}-1 & ;\;if\;DEA\;efficiency\;score<1 \end{array}\right.$$ (3)

The set of explanatory variables included binary variables representing each potential determinant (COMMER, ED, ICU). The model included variables based on the literature review and two control variables representing gross profit/loss of the hospital (GROSS.PROFIT) and equities (EQ). The set of variables developed, based on the literature review, included the shares of doctors (DOC.SHARE) [10], nurses (NURSE.SHARE), and the laboratory diagnostic staff (defined as the sum of laboratory diagnosticians and medical analytics technicians) (DIAG.SHARE); in total staff numbers.

It is important to note that the availability of diagnostic laboratories on-site allows for quicker completion of a correct diagnosis and, in turn, quicker and more effective treatment (compare [45,46]). It could therefore be hypothesized that laboratory diagnostic capabilities improve the overall efficiency of a hospital even if they also mean higher costs. As there is no specific data about whether there is a diagnostic laboratory in the hospital, it was assumed that the hospital has diagnostic capabilities if it employs laboratory diagnosticians or medical analytics technicians.

Variables representing ratios of nurses and doctors to the number of beds (NURSE.BEDS and DOC.BEDS) were also included in the model. The last two explanatory variables were defined as the share of ED and ICU beds in the total number of beds (ED. SHARE and ICU.SHARE, respectively).

The model is specified as:

$$\begin{array}{l} ineff_{i}=\alpha_{0}+\alpha_{1}ED_{i}+\alpha_{2}ICU_{i}+\alpha_{3}{COMMER}_{i}+\alpha_{4}DIAG_{i}+\alpha_{5}GROSS.PROFIT_{i}+\alpha_{6}EQ_{i}\\ \;+\alpha_{7}DOC.SHARE_{i}+\alpha_{8}NURSE.SHARE_{i}+\alpha_{9}DIAG.SHARE+\alpha_{10}ED.SHARE_{i}\\ \;+\alpha_{11}ICU.SHARE_{i}+\alpha_{12}DOC.B{EDS}_{i}+\alpha_{13}NURSE.B{EDS}_{i}+\alpha_{14}{BIG}_{i}\\ \;+\alpha_{15}{SMALL}_{i}+\epsilon_{i} \end{array}$$

Where:

ineff–inefficiency score defined by formula (3),

ED–ED dummy variable (1 if a hospital has an ED in its structure, 0 otherwise),

ICU–ICU dummy variable (1 if a hospital has an ICU in its structure, 0 otherwise),

COMMER–commercialized dummy variable (1 if it is a commercialized hospital, 0 otherwise),

DIAG–diagnostics dummy variable (1 if a hospital has on-site diagnostics, 0 otherwise),

GROSS.PROFIT–gross profit/loss,

EQ–equity,

DOC.SHARE–share of doctors in all staff,

NURSE.SHARE–share of nurses in all staff,

DIAG.SHARE–share of laboratory diagnostic staff in all staff,

ED.SHARE–share of ED beds in the total number of beds,

ICU.SHARE–share of ICU beds in the total number of beds,

SMALL–small number of beds dummy variable (less than mean minus standard deviation, calculated separately for each subgroup of hospitals),

BIG–big number of beds dummy variable (less than mean minus standard deviation, calculated separately for each subgroup of hospitals),

NURSE.BEDS–ratio of nurses to the number of beds,

DOC.BEDS–ratio of doctors to the number of beds.

Tobit models were estimated with robust errors, using a general-to-specific approach, sequentially eliminating variables with the highest p-values and α = 0.05. Estimation was done in gretl software (ver. 2018c) [47].

### Data

The data used in the analysis was from a voluntary questionnaire prepared by the Polish Association of Employers of Polish county Hospitals (OZPSP–Ogólnopolski Związek Pracodawców Szpitali Powiatowych), which was completed by around 110 Polish county hospitals during summer 2019. Cases of ambiguous answers were removed from the database. There was no direct question in the questionnaire regarding whether an ED or ICU functions in the hospital. It was therefore assumed that the hospital has these units if it reports a non-zero number of emergency beds or ICU beds, respectively. If the hospital reported zero beds in either category, it was assumed that the respective unit does not exist. Descriptive statistics for the dataset used in the study are presented in Table 2.

**Table 2:** Descriptive statistics for output, inputs and explanatory variables by subgroup.

	Total number of beds	Contract with NHF [thousands of zł]	Doctors [full-time equivalents]	Nurses [full-time equivalents]	Patient-days total	Materials (medical) [thousands of zł]	Materials (nonmedical) [thousands of zł]	Outsourcing (medical) [thousands of zł]	Outsourcing (nonmedical) [thousands of zł]	Energy [thousands of zł]	Service workers [full-time equivalents]	Administration staff [full-time equivalents]	Fixed assets [thousands of zł]	Current assets [thousands of zł]	doctors.share	nurses.share	diag.share	ED.share	ICU.share	doc.beds	nur.beds	EQ [thousands of zł]	Gross profit/loss [thousands of zł]	ICU beds	non-ICU beds	diag	ED	ICU	COMMER
	**General approach**																												
**min**	57	598.9	0.67	56.25	1.295	21.04	21.15	4.539	40.18	17.25	3	7.15	1.771	116.5	0.003	0.1557	0	0	0	0.0027	0.374	-5386.1	-1388.37			0: 44	0: 156	0: 46	0: 236
$\bar{x}$	261.7	4475.6	128.4	397.91	6.423	651.88	128.99	1076.691	329.79	100.12	88.99	63.74	3231.756	988.1	0.1378	0.4235	0.0298	0.0123	0.0185	0.4676	1.5144	1241.8	-88.98			1: 212	1: 154	1: 264	1: 74
**Q2**	237	3569.2	58.84	192.41	5.646	410.83	93.51	931.433	242.02	72.3	33.81	30.84	2402.807	694.6	0.142	0.4157	0.0326	0	0.0185	0.2804	0.8154	773.9	-38.45			NA: 54	NA: 0	NA: 0	NA: 0
**max**	599	15813.9	2379.83	6492	15.706	3247.81	855.09	3517.213	1680.48	481.95	1812	1104	18983.215	4228.1	0.2847	0.6266	0.0606	0.0524	0.0748	4.5182	12.2955	15222.1	607.67						
**NA**	0	0	0	0	0	0	0	0	0	0	0	0	0	0	54	54	54	0	0	0	0	0	0						
	**Detailed approach. hospitals with an ICU**																												
**min**		598.9	0.67	82	1.95	21.04	21.15	68.54	66.69	17.25	3	9.5	1.771	161.7															
$\bar{x}$		4899	138.21	433.8	7.032	732.3	141.76	1170.57	358.19	110.21	95.96	67.9	3516.337	1101.8															
**Q2**		4146	62.85	206.1	6.602	492.54	106.35	1054.52	256.31	80.5	46.16	33.1	2797.245	774															
**max**		15813.9	2379.83	6492	15.706	3247.81	855.09	3517.21	1680.48	481.95	1812	1104	18983.215	4228.1															
**NA**		0	0	0	0	0	0	0	0	0	0	0	0	0															
	**Detailed approach: hospitals with no ED**																												
**min**	57	913.6	0.67	56.25	1.295	21.04	21.15	4.539	40.18	17.25	3	7.15	1.771	116.5															
$\bar{x}$	215	3113.3	56.4	220.47	5.302	364.23	90.23	791.715	228.04	72.48	59.21	36.03	1794.722	663.8															
**Q2**	177.5	2738.5	44.33	142.33	4.186	315.09	73.02	729.265	199	56.9	20.55	25.01	1497.677	436.9															
**max**	417	8002.3	464.75	1635.12	12.269	1554.78	355.06	2027.838	603.75	213.45	676.08	270.07	7583.112	3220.3															
**NA**	0	0	0	0	0	0	0	0	0	0	0	0	0	0															
	**Detailed approach: public hospitals**																												
**min**	57	598.9	11.75	56.25	1.295	21.04	21.15	4.539	40.18	17.25	3	7.15	93.17	161.7															
$\bar{x}$	267.8	4824.5	147.93	430.95	6.59	726.77	144.62	1107.091	346.88	104.39	89.94	68.51	3493.83	1081.3															
**Q2**	242.5	3861.2	63.7	203.65	5.816	444.2	109.05	928.483	242.02	73.08	32.94	30.94	2704.57	756.6															
**max**	599	15813.9	2379.83	6492	15.706	3247.81	855.09	3517.213	1680.48	481.95	1812	1104	18983.22	4228.1															
**NA**	0	0	0	0	0	0	0	0	0	0	0	0	0	0															
	**Traditional approach: all hospitals**																												
**min**	57		0.67	56.25	1.295										0.003	0.1557	0	0	0	0.0027	0.374	-5386.141	-1388.37			0: 47	0: 162	0: 48	0: 253
$\bar{x}$	257.1		124.63	381.94	6.271										0.1395	0.4236	0.0291	0.0133	0.0187	0.4608	1.4708	1156.06	-90.94			1: 216	1: 170	1: 284	1: 79
**Q2**	229.5		58.47	190	5.457										0.1429	0.4173	0.0322	0.0082	0.0188	0.2832	0.8211	696.458	-38.23			NA: 69	NA: 0	NA: 0	NA: 0
**max**	599		2379.83	6492	15.706										0.2847	0.6266	0.0606	0.0524	0.0748	4.5182	12.2955	15222.083	607.67						
**NA**	0		0	0	0										69	69	69	0	0	0	0	1	1						
	**Traditional approach: public hospitals**																												
**min**	57		11.75	56.25	1.295																								
$\bar{x}$	262.4		142.22	411.93	6.429																								
**Q2**	237		62.95	198	5.684																								
**max**	599		2379.83	6492	15.706																								
**NA**	0		0	0	0																								
	**Traditional approach: commercial hospitals**																												
**min**	127		0.67	74	1.746																								
$\bar{x}$	240.1		68.3	285.9	5.764																								
**Q2**	203		50.31	172.2	4.972																								
**max**	417		464.75	1635.1	12.269																								
**NA**	0		0	0	0																								
	**Traditional approach: hospitals with no ED**																												
**min**	57		0.67	56.25	1.295																								
$\bar{x}$	212.6		55.83	217.1	5.217																								
**Q2**	173		44.83	142.83	4.087																								
**max**	417		464.75	1635.12	12.269																								
**NA**	0		0	0	0																								
	**Traditional approach: hospitals with an ED**																												
**min**	61		12.9	82	1.457																								
$\bar{x}$	299.5		190.2	539	7.275																								
**Q2**	270.5		85.17	234.5	6.91																								
**max**	599		2379.83	6492	15.706																								
**NA**	0		0	0	0																								
	**Traditional approach: hospitals with an ICU**																												
**min**	88		0.67	82	1.95																								
$\bar{x}$	277.1		133.75	414.8	6.845																								
**Q2**	262		62.75	203.7	6.195																								
**max**	599		2379.83	6492	15.706																								
**NA**	0		0	0	0																								

$\bar{x}$ – mean, Q2 –median, NA–missing values, dummies for big and small hospitals are not presented, since in the Tobit models they are calculated for the sample that does not include missing observations. Explanatory variables are presented only for subgroups from which regression models were estimated.

Depending on the group of hospitals, the median number of beds ranged from 173 to 270.5. Median employment ranged from 44.33 to 85.17 full-time equivalents for doctors, and from 142.33 to 234.5 full-time equivalents for nurses. These translated into median shares of doctors and nurses in the whole staff equal to 0.1279–0.1484 and 0.3998–0.4314, respectively. Median shares of diagnostics staff were lower and ranged from 0.0279–0.03304. Median patient-days ranged from 40,870 to 69,100. Statistically, hospitals had an average of 5 ICU beds—the median share of ICU beds ranging from 0.0162 to 0.0207 and the median share of ED beds on a level 0–0.0233.

Table 3 presents the numbers of hospitals that are included in the DEA analysis after constructing the set of hospitals, as previously described. Subgroups containing numbers of hospitals that total less than 5 are not included. Following Golany and Roll [42], it is reasonable to conclude that the total number of hospitals in each subgroup is high enough to conduct DEA analysis.

**Table 3:** Numbers of hospitals by year, subgroup and approach.

Group or subgroup			2015	2016	2017	2018
***General* approach**						
General	Commercialized:	0	56	59	60	61
		1	18	18	19	19
	ED:	0	38	39	40	39
		1	36	38	39	41
	ICU:	0	10	12	12	12
		1	64	65	67	68
***Detailed* approach**						
Public hospitals	ED:	0	24	25	25	24
		1	32	34	35	37
	ICU:	0	6	8	8	8
		1	50	51	52	53
Hospitals with no ED	ICU:	0	10	10	10	10
		1	28	29	30	29
	Commercialized:	0	24	25	25	24
		1	14	14	15	15
Hospitals with ICU	ED:	0	28	29	30	29
		1	36	36	37	39
	Commercialized:	0	50	51	52	53
		1	14	14	15	15
***Traditional* approach**						
General	ED:	0	40	40	41	41
		1	41	42	42	45
	ICU:	0	11	12	12	13
		1	70	70	71	73
	Commercialized:	0	62	63	63	65
		1	19	19	20	21
Public hospitals	ED:	0	26	26	26	26
		1	36	37	37	39
	ICU:	0	7	8	8	9
		1	55	55	55	56
Hospitals with ED	Commercialized:	0	36	37	37	39
		1	5	5	5	6
Hospitals with no ED	ICU:	0	10	10	10	11
		1	30	30	31	30
	Commercialized:	0	26	26	26	26
		1	14	14	15	15
Hospitals with ICU	ED:	0	30	30	31	30
		1	40	40	40	43
	Commercialized:	0	55	55	55	56
		1	15	15	16	17
Commercialized hospitals	ED:	0	14	14	15	15
		1	5	5	5	6

## Results

### The *general* approach

The results of efficiency analysis in the whole group of hospitals are presented in Table 4. As shown by the results of Chi2 test, the form of ownership did not have a significant impact on the efficiency of a hospital. Both the means and medians point to higher efficiency in commercialized hospitals, but the differences in means were not statistically significant. For commercialized hospitals, the means were higher than medians, which leads to the conclusion that there were outliers in this group, i.e. commercialized hospitals exist with relatively very high efficiency levels. On the other hand, ED in the structure lowered the efficiency of a hospital (in all years, as shown by the results of the Kruskall-Wallis test) and affected whether a hospital was efficient in 2015–2017. ICU had a significant impact on whether a hospital is considered efficient for the whole period, as shown by the results of Chi2 test, while the difference in mean efficiency between hospitals with and without ICU was significant only in 2015. However, the comparison of means and medians gives inconclusive results. Means point to a higher efficiency of hospitals with an ICU, while median efficiency in this group was lower than in the group of hospitals with no ICU.

**Table 4:** Results of hospital efficiency with respect to ownership, ED and ICU (*general* approach).

Result Year	No. of efficient hospitals (no. of inefficient hospitals)		Median efficiency		Mean efficiency		Chi2 test		KW test	
**Form of ownership**										
	**Public**	**Commercialized**	**Commercialized**	**Public**	**Commercialized**	**Public**	**statistics**	**p-value**	**statistics**	**p-value**
2015	27(28)	14(4)	1.5329	0.9989	7.8463	1.4404	3.4427	0.0635	3.8344	0.0502
2016	32(26)	14(4)	1.2795	1.0489	7.6448	1.5326	2.0681	0.1504	3.0525	0.0806
2017	33(26)	15(4)	1.3021	1.0963	7.5039	1.3866	2.3175	0.1279	2.8293	0.0926
2018	34(26)	14(5)	1.3336	1.042	6.3203	1.3085	1.1117	0.2917	3.2021	0.0735
**ICU**										
	**No ICU**	**ICU**	**ICU**	**No ICU**	**ICU**	**No ICU**	**statistics**	**p-value**	**statistics**	**p-value**
2015	10(0)	31(32)	0.9989	1.338	3.1006	2.511	7.0983	0.0077	4.1516	0.0416
2016	11(1)	35(29)	1.0489	1.2573	3.089	2.4001	4.3395	0.0372	2.6372	0.1044
2017	11(1)	37(29)	1.0959	1.251	3.0808	1.755	4.0385	0.0445	1.8046	0.1792
2018	11(1)	37(30)	1.05	1.263	2.7073	1.434	4.2435	0.0394	1.7194	0.1898
**ED**										
	**No ED**	**ED**	**ED**	**No ED**	**ED**	**No ED**	**statistics**	**p-value**	**statistics**	**p-value**
2015	29(9)	12(23)	0.8541	1.228	1.1586	4.734	11.421	0.0007	12.958	0.0003
2016	31(8)	15(22)	0.8998	1.248	1.1397	4.7264	10.479	0.0012	12.595	0.0004
2017	30(10)	18(20)	0.9537	1.3214	1.2212	4.4494	5.173	0.0229	10.233	0.0014
2018	28(11)	20(20)	1.0116	1.3296	1.2242	3.8367	3.0732	0.0796	8.8862	0.0029

The first number given in columns 1–2 is the number of efficient hospitals in a respective subgroup. Numbers in parentheses represent the number of inefficient hospitals in the same subgroup. The sum of these two values may be different from the number in Table 2 because the model can be infeasible for some hospitals. Both the KW and Chi-square tests are conducted with 1 degree of freedom. α = 0.05 is assumed for the interpretation.

### The *detailed* approach

#### ICU

The results of efficiency analysis in the subgroup of hospitals with an ICU are presented in Table 5. The results show that the form of ownership had no significant impact on efficiency. On the other hand, the presence of an ED in a hospital significantly lowered mean efficiency and influenced whether the hospital was considered efficient or not. Comparison of means and medians leads to the conclusion that there were high-efficient outliers within the subgroups of commercialized hospitals and hospitals with no ED.

**Table 5:** The efficiency of hospitals with an ICU with respect to the form of ownership and ED (*detailed* approach).

Result Year	No. of efficient hospitals (no. of inefficient hospitals)		Median efficiency		Mean efficiency		Chi2 test		KW test	
**Form of ownership**										
	**Public**	**Commercialized**	**Commercialized**	**Public**	**Commercialized**	**Public**	**statistics**	**p-value**	**statistics**	**p-value**
2015	25(24)	10(4)	1.5189	1.0061	9.9584	1.3331	1.1032	0.2936	1.4967	0.2212
2016	28(22)	10(4)	1.3519	1.0152	9.5909	1.423	0.5345	0.4647	2.5329	0.1115
2017	26(25)	12(3)	1.3296	1.0466	9.252	1.3913	2.8964	0.0888	2.4598	0.1168
2018	28(24)	12(3)	1.4523	1.0563	7.6871	1.3639	2.3121	0.1284	2.6389	0.1043
**ED**										
	**No ED**	**ED**	**ED**	**No ED**	**ED**	**No ED**	**statistics**	**p-value**	**statistics**	**p-value**
2015	21(7)	14(21)	0.9393	1.4415	1.2394	5.7629	6.3651	0.0116	8.762	0.0031
2016	23(6)	15(20)	0.9292	1.4457	1.2102	5.623	7.2911	0.0069	10.52	0.0012
2017	22(8)	16(20)	0.9549	1.399	1.2921	5.4407	4.4708	0.0345	9.3149	0.0023
2018	22(7)	18(20)	0.9927	1.477	1.2676	4.761	4.4293	0.0353	7.6803	0.0056

The first number given in columns 1–2 is the number of efficient hospitals in a respective subgroup. Numbers in parentheses represent the number of inefficient hospitals in the same subgroup. The sum of these two values may be different from the number in Table 3 because the model can be infeasible for some hospitals. Both the KW and Chi-square tests are conducted with 1 degree of freedom. α = 0.05 is assumed for the interpretation.

#### No-ED

Results presented in Table 6 show that in cases of hospitals with no ED, neither the form of ownership nor ICU had a significant impact on the efficiency (both on whether a hospital is considered efficient and on the mean efficiency). The results suggest the presence of high-efficient outliers in the following subgroups: commercialized hospitals, hospitals with an ICU (in all years) and hospitals without an ICU (in 2015–2016).

**Table 6:** Efficiency of hospitals without an ED with respect to the form of ownership and ICU (*detailed* approach).

Result Year	No. of efficient hospitals (no. of inefficient hospitals)		Median efficiency		Mean efficiency		Chi2 test		KW test	
**Form of ownership**										
	**Public**	**Commercialized**	**Commercialized**	**Public**	**Commercialized**	**Public**	**statistics**	**p-value**	**statistics**	**p-value**
2015	16(8)	12(1)	1.59	1.1126	10.184	2.1612	1.7799	0.1822	2.8431	0.0918
2016	18(7)	12(1)	1.4041	1.1566	9.7316	2.7686	1.0762	0.2996	2.1361	0.1439
2017	17(8)	12(2)	1.4606	1.1817	9.4845	1.6882	0.694	0.4048	1.05	0.3055
2018	16(8)	11(3)	1.3995	1.1969	7.835	1.4257	0.1679	0.682	0.8242	0.364
**ICU**										
	**No ICU**	**ICU**	**ICU**	**No ICU**	**ICU**	**No ICU**	**statistics**	**p-value**	**statistics**	**p-value**
2015	10(0)	18(9)	1.1876	1.338	5.6379	3.204	2.78	0.0955	0.5661	0.4518
2016	9(1)	21(7)	1.1738	1.2795	5.4202	4.396	0.2991	0.5844	0.0538	0.8165
2017	9(1)	20(9)	1.3578	1.295	5.369	1.929	0.7987	0.3715	0.001	0.9743
2018	9(1)	18(10)	1.2756	1.287	4.6081	1.488	1.2836	0.2572	0.0538	0.8165

The first number given in columns 1–2 is the number of efficient hospitals in a respective subgroup. Numbers in parentheses represent the number of inefficient hospitals in the same subgroup. The sum of these two values may be different from the number in Table 2 because the model can be infeasible for some hospitals. Both the KW and Chi-square tests are conducted with 1 degree of freedom. α = 0.05 is assumed for the interpretation.

#### Public hospitals

The results of the efficiency analysis of public hospitals are presented in Table 7. As shown in the top part of the table, ICU was not a significant determinant of the efficiency level. Differences in mean efficiency between hospitals with and without an ICU unit were not significant. Furthermore, ICU did not affect whether a hospital is efficient, as shown by the results of chi2 test.

**Table 7:** Efficiency of public hospitals with respect to ED and ICU (*detailed* approach).

Result Year	No. of efficient hospitals (no. of inefficient hospitals)		Median efficiency		Mean efficiency		Chi2 test		KW test	
**ICU**										
	**No ICU**	**ICU**	**ICU**	**No ICU**	**ICU**	**No ICU**	**statistics**	**p-value**	**statistics**	**p-value**
2015	6(0)	27(22)	1.0857	1.326	1.3526	3.231	2.8139	0.0935	2.4519	0.1174
2016	8(0)	29(21)	1.0962	1.25	1.4193	2.985	3.6056	0.0576	2.3512	0.1252
2017	8(0)	32(19)	1.1012	1.271	1.3794	2.022	2.8553	0.0911	2.0078	0.1565
2018	8(0)	33(19)	1.1104	1.282	1.3672	1.554	2.7558	0.0969	1.5908	0.2072
**ED**										
	**No ED**	**ED**	**ED**	**No ED**	**ED**	**No ED**	**statistics**	**p-value**	**statistics**	**p-value**
2015	18(6)	15(16)	0.9663	1.2385	1.2659	1.9343	2.9601	0.0853	5.4058	0.0201
2016	19(6)	18(15)	1.092	1.2574	1.2536	2.1393	1.9819	0.1592	5.2192	0.0223
2017	20(5)	20(14)	1.0718	1.3687	1.2933	1.7022	2.0687	0.1504	5.0155	0.0251
2018	19(5)	22(14)	1.0664	1.243	1.3081	1.518	1.4153	0.2342	3.6724	0.0553

The first number given in columns 1–2 is the number of efficient hospitals in a respective subgroup. Numbers in parentheses represent the number of inefficient hospitals in the same subgroup. The sum of these two values may be different from the number in Table 2 because the model can be infeasible for some hospitals. Both the KW and Chi-square tests are conducted with 1 degree of freedom. α = 0.05 is assumed for the interpretation.

Efficiency was higher in the group of hospitals that do not have an ED in their structure and the differences in mean efficiency were statistically significant in 2015–2017, as shown by the results of Kruskall-Wallis test. However, whether a hospital was considered efficient was not significantly influenced by this determinant.

### The *traditional* approach

#### Whole group

The results of the efficiency analysis of all types of hospitals are presented in Table 8. As shown in the top part of the table, although mean efficiency was higher for commercialized hospitals, the difference was insignificant. Results of the Chi2 test lead to the conclusion that the form of ownership affects whether a hospital is considered efficient only in 2016–2017. Comparison of mean and median efficiency levels leads to the conclusion that high-efficient outliers exist within the group of commercialized hospitals. As shown in the middle part of Table 8, the presence of an ICU within the hospital structure had no significant effect on efficiency (both on whether a hospital is considered efficient and on the mean efficiency). When ED is considered (bottom part of Table 7), general conclusions are similar to those of the previous determinant. The difference in means was not significant and an ED in the hospital structure did not significantly influence whether a hospital was considered efficient or not.

**Table 8:** Efficiency of hospitals with respect to the form of ownership, ED and ICU (*traditional* approach).

Result Year	No. of efficient hospitals (no. of inefficient hospitals)		Median efficiency		Mean efficiency		Chi2 test		KW test	
**Form of ownership**										
	**Public**	**Commercialized**	**Commercialized**	**Public**	**Commercialized**	**Public**	**statistics**	**p-value**	**statistics**	**p-value**
2015	4(57)	4(15)	0.7626	0.7687	1.8071	0.8163	1.9634	0.1612	0.1112	0.7387
2016	4(58)	5(14)	0.7442	0.7608	1.3595	0.808	3.9731	0.0462	0.4179	0.518
2017	4(58)	6(14)	0.7932	0.7651	1.6938	0.8086	5.7864	0.0162	0.3275	0.5671
2018	5(59)	4(17)	0.7706	0.7489	1.8991	0.8233	1.0885	0.2968	0.3614	0.5477
**ICU**										
	**No ICU**	**ICU**	**ICU**	**No ICU**	**ICU**	**No ICU**	**statistics**	**p-value**	**statistics**	**p-value**
2015	1(10)	7(62)	0.7664	0.8177	1.0794	0.8776	0	1	0.246	0.6199
2016	1(11)	8(61)	0.7594	0.775	0.9552	0.8351	0	1	0.0855	0.7699
2017	1(11)	9(61)	0.7692	0.7274	1.0639	0.795	0	1	0.3182	0.5727
2018	1(12)	8(64)	0.7489	0.7706	1.1394	0.8104	0	1	0.0382	0.8451
**ED**										
	**No ED**	**ED**	**ED**	**No ED**	**ED**	**No ED**	**statistics**	**p-value**	**statistics**	**p-value**
2015	5(35)	3(37)	0.7675	0.7742	0.798	1.3052	0.1389	0.7094	0.9633	0.3263
2016	6(34)	3(38)	0.7594	0.7575	0.7983	1.0799	0.5572	0.4554	0.6447	0.422
2017	7(34)	3(38)	0.766	0.7642	0.8144	1.2346	1.025	0.3113	0.0398	0.842
2018	6(35)	3(41)	0.7354	0.8028	0.8234	1.3742	0.6683	0.4136	0.5654	0.4521

The first number given in columns 1–2 is the number of efficient hospitals in a respective subgroup. Numbers in parentheses represent the number of inefficient hospitals in the same subgroup. The sum of these two values may be different from the number in Table 3 because the model can be infeasible for some hospitals. Both the KW and Chi-square tests are conducted with 1 degree of freedom. α = 0.05 is assumed for the interpretation.

#### Commercialized

In the group of commercialized hospitals, both mean and median efficiency was higher for hospitals that did not have an ED (Table 9), and for the years 2015–2016 and 2018 differences in means were statistically significant. On the other hand, an ED did not significantly affect whether a hospital is considered efficient or not, as shown by the results of the Chi2 test.

**Table 9:** Efficiency of commercialized hospitals with respect to an ED (*traditional* approach).

Result Year	No. of efficient hospitals (no. of inefficient hospitals)		Median efficiency		Mean efficiency		Chi2 test		KW test	
**ED**										
	**No ED**	**ED**	**ED**	**No ED**	**ED**	**No ED**	**statistics**	**p-value**	**statistics**	**p-value**
2015	6(7)	0(5)	0.8034	0.9982	0.8154	2.5277	1.6962	0.1928	6.8235	0.009
2016	6(7)	0(5)	0.8019	0.9852	0.8426	1.8428	1.6962	0.1928	6.3182	0.012
2017	8(6)	1(4)	0.8174	1.012	0.8828	2.5316	0.8211	0.3649	3.0943	0.0786
2018	6(8)	2(4)	0.7914	0.96	0.8587	2.8541	0	1	3.9184	0.0478

The first number given in columns 1–2 is the number of efficient hospitals in a respective subgroup. Numbers in parentheses represent the number of inefficient hospitals in the same subgroup. The sum of these two values may be different from the number in Table 3 because the model can be infeasible for some hospitals. Both KW and Chi-square tests are conducted with 1 degree of freedom. α = 0.05 is assumed for the interpretation.

#### Public

The results for public hospitals are presented in Table 10. They lead to the conclusion that within this group of hospitals there are no differences in efficiency for both potential determinants, even though both mean and median efficiency scores were usually higher in cases of hospitals with no ED or ICU.

**Table 10:** Efficiency of public hospitals with respect to an ED and ICU (*traditional* approach).

Result Year	No. of efficient hospitals (no. of inefficient hospitals)		Median efficiency		Mean efficiency		Chi2 test		KW test	
**ICU**										
	**No ICU**	**ICU**	**ICU**	**No ICU**	**ICU**	**No ICU**	**statistics**	**p-value**	**statistics**	**p-value**
2015	2(5)	5(49)	0.7877	0.9344	0.8338	1.0035	0.7711	0.3799	1.6057	0.2051
2016	2(6)	6(48)	0.7801	0.8536	0.8362	0.9351	0.2794	0.5971	0.5097	0.4753
2017	1(7)	7(47)	0.7869	0.8151	0.8456	0.8685	0	1	0.0441	0.8337
2018	1(8)	6(49)	0.7948	0.8449	0.8559	0.8741	0	1	0.3246	0.5689
**ED**										
	**No ED**	**ED**	**ED**	**No ED**	**ED**	**No ED**	**statistics**	**p-value**	**statistics**	**p-value**
2015	4(22)	3(32)	0.7937	0.8181	0.8472	0.8614	0.176	0.6749	0.0417	0.8382
2016	4(22)	4(32)	0.7775	0.8156	0.8505	0.8469	0.0124	0.9113	0.2939	0.5878
2017	3(23)	5(31)	0.7828	0.8151	0.8706	0.818	0	1	0.0033	0.9545
2018	4(22)	3(35)	0.7743	0.8093	0.8657	0.8478	0.2864	0.5925	0.0988	0.7532

The first number given in columns 1–2 is the number of efficient hospitals in a respective subgroup. Numbers in parentheses represent the number of inefficient hospitals in the same subgroup. The sum of those two values may be different from the number in Table 3 because the model can be infeasible for some hospitals. Both KW and Chi-square tests are conducted with 1 degree of freedom. α = 0.05 is assumed for the interpretation.

#### ED

The results for hospitals with an ED are presented in Table 11. For this group, the form of ownership had no impact on whether the hospital was considered efficient, nor on the efficiency score, as shown by the results of the KW and Chi2 tests.

**Table 11:** Efficiency of hospitals with an ED with respect to the form of ownership (*traditional* approach).

Result Year	No. of efficient hospitals (no. of inefficient hospitals)		Median efficiency		Mean efficiency		Chi2 test		KW test	
**Form of ownership**										
	**Public**	**Commercialized**	**Commercialized**	**Public**	**Commercialized**	**Public**	**statistics**	**p-value**	**statistics**	**p-value**
2015	9(26)	0(5)	0.8076	0.864	0.8093	0.9918	0.512	0.4743	1.3585	0.2438
2016	9(27)	1(4)	0.8481	0.855	0.8359	1.0013	0	1	0.573	0.4491
2017	8(28)	1(4)	0.8397	0.8457	0.8229	0.9966	0	1	0.5143	0.4733
2018	9(29)	1(5)	0.8274	0.8751	0.8923	1.0099	0	1	0.4222	0.5158

The first number given in columns 1–2 is the number of efficient hospitals in a respective subgroup. Numbers in parentheses represent the number of inefficient hospitals in the same subgroup. The sums of these two values may be different from the number in Table 3 because the model can be infeasible for some hospitals. Both the KW and Chi-square tests are conducted with 1 degree of freedom. α = 0.05 is assumed for the interpretation.

#### No-ED

In the subgroup of hospitals with no ED (Table 12), none of the potential determinants was found to significantly influence the efficiency. There was only one exception—the form of ownership affected whether a hospital was considered efficient in 2017.

**Table 12:** Efficiency of hospitals without ED with respect to the form of ownership and ICU (*traditional* approach).

Result Year	No. of efficient hospitals (no. of inefficient hospitals)		Median efficiency		Mean efficiency		Chi2 test		KW test	
**Form of ownership**										
	**Public**	**Commercialized**	**Commercialized**	**Public**	**Commercialized**	**Public**	**statistics**	**p-value**	**statistics**	**p-value**
2015	3(23)	3(11)	0.8748	0.7612	2.3081	0.8082	1.7063	0.1915	1.9607	0.1614
2016	1(25)	5(9)	0.84	0.7646	1.6435	0.7981	3.4699	0.0625	1.8781	0.1705
2017	1(25)	4(9)	0.7952	0.789	2.2354	0.7965	4.9644	0.0259	0.985	0.321
2018	1(25)	3(10)	0.812	0.8047	2.6008	0.8252	0.1379	0.7104	0.5436	0.461
**ICU**										
	**No ICU**	**ICU**	**ICU**	**No ICU**	**ICU**	**No ICU**	**statistics**	**p-value**	**statistics**	**p-value**
2015	1(9)	3(26)	0.7708	0.7878	1.4553	0.8815	0	1	0.0507	0.8219
2016	1(9)	4(25)	0.767	0.775	1.1598	0.8484	0	1	0.5472	0.4594
2017	1(9)	5(25)	0.7978	0.7306	1.4669	0.7995	0	1	0.7112	0.399
2018	1(10)	5(24)	0.852	0.7706	1.6862	0.8151	0.0221	0.8817	1.0297	0.3102

The first number given in columns 1–2 is the number of efficient hospitals in a respective subgroup. Numbers in parentheses represent the number of inefficient hospitals in the same subgroup. The sum of those two values may be different from the number in Table 3 because the model can be infeasible for some hospitals. Both the KW and Chi-square tests are conducted with 1 degree of freedom. α = 0.05 is assumed for the interpretation.

#### ICU

In the subgroup of hospitals with an ICU, the form of ownership did not impact the mean efficiency score, nor whether a hospital can be considered efficient, as shown in Table 13. Comparison of means and medians leads to the conclusion that there were high-efficient outliers within the subgroup of commercialized hospitals. On the other hand, the presence of an ED in a hospital structure significantly decreased the mean efficiency score as shown by the results of KW test. ED also affected whether a hospital was considered efficient, but only in 2016.

**Table 13:** Efficiency of hospitals with an ICU with respect to the form of ownership and ED (*traditional* approach).

Result Year	No. of efficient hospitals (no. of inefficient hospitals)		Median efficiency		Mean efficiency		Chi2 test		KW test	
**Form of ownership**										
	**Public**	**Commercialized**	**Commercialized**	**Public**	**Commercialized**	**Public**	**statistics**	**p-value**	**statistics**	**p-value**
2015	8(46)	4(11)	0.8861	0.8391	2.2531	0.8911	0.471	0.4925	0.1543	0.6945
2016	7(47)	5(10)	0.8551	0.8164	1.6049	0.8952	2.1209	0.1453	1.0971	0.2949
2017	7(47)	6(10)	0.9081	0.8242	1.9958	0.8833	3.4254	0.0642	0.3961	0.5291
2018	8(47)	4(13)	0.9188	0.8267	2.2283	0.8924	0.2464	0.6196	0.2743	0.6005
**ED**										
	**No ED**	**ED**	**ED**	**No ED**	**ED**	**No ED**	**statistics**	**p-value**	**statistics**	**p-value**
2015	8(22)	4(35)	0.7988	0.8917	0.8522	1.6227	2.1388	0.1436	10.602	0.0011
2016	9(21)	3(36)	0.7914	0.9155	0.8599	1.296	4.4233	0.0355	11.569	0.0007
2017	9(22)	4(35)	0.7926	0.9191	0.8594	1.4875	2.8804	0.0897	6.4321	0.0112
2018	8(22)	4(38)	0.7871	0.9211	0.8693	1.6817	2.5714	0.1088	7.0827	0.0078

The first number given in columns 1–2 is the number of efficient hospitals in a respective subgroup. Numbers in parentheses represent the number of inefficient hospitals in the same subgroup. The sum of these two values may be different from the number in Table 3 because the model can be infeasible for some hospitals. Both the KW and Chi-square tests are conducted with 1 degree of freedom. α = 0.05 is assumed for the interpretation.

### Regression models

The results of Tobit regression for the inefficiency score, calculated in the *general* approach, are presented in Table 14. The set of determinants changes in 2015–2018 and only two variables were significant for the whole period—dummy for small hospitals (SMALL), which had a negative relationship with inefficiency, and the share of ED beds (ED.SHARE), which had a positive relationship with inefficiency. EQ had a positive relationship with inefficiency, which may lead to the conclusion that bigger hospitals (in terms of capital) are less efficient. This impact is significant but very small. The ratio of nurses to beds (NURS.BEDS) had a positive relationship with inefficiency, while the share of nurses in the total staff (NURSE.SHARE) had a negative effect, leading to a decrease in inefficiency. The presence of the ICU in the hospital (ICU) was significant only in 2015 and—as expected—the parameter was positive. It showed that an ICU within the hospital structure significantly lowers the efficiency. The impact of the DOC.BEDS variable representing the ratio of doctors to beds was inconclusive; positive in 2015, and negative in 2017.

**Table 14:** Results of Tobit regression of inefficiency on hospital characteristics (*general* approach).

Year Variable	2015		2016		2017		2018	
	Parameter	p-value	Parameter	p-value	Parameter	p-value	Parameter	p-value
	(std.error)		(std.error)		(std.error)		(std.error)	
const	-1.08045 (0.13743)	p<0.0001	-0.09532 (0.05074)	0.0603	0.22514 (0.13589)	0.0976	-0.13121 (0.0538)	0.0147
EQ	0.00002 (0.00001)	0.0304			0.00002 (0.00001)	0.0003		
ED.SHARE	6.36996 (2.25183)	0.0047	7.21386 (2.10106)	0.0006	5.94185 (2.17655)	0.0063	5.59281 (2.06344)	0.0067
DOC.BEDS	0.03975 (0.01991)	0.0459			-0.21853 (0.09251)	0.0182		
ICU	1.00654 (0.12096)	p<0.0001						
SMALL	-1.0149 (0.12035)	p<0.0001	-1.2265 (0.14787)	p<0.0001	-1.79895 (0.43057)	p<0.0001	-1.29687 (0.17739)	p<0.0001
NURS.BEDS			0.0219 (0.0077)	0.0045	0.09927 (0.03491)	0.0045	0.02566 (0.01112)	0.021
NURSE.SHARE					-0.90779 (0.32298)	0.0049		
sima (SE)	0.186997	(0.02414)	0.192803	(0.02727)	0.169572	(0.02711)	0.207644	(0.02647)
chi2	126.4671	p<0.0001	97.4489	p<0.0001	83.89358	p<0.0001	86.4735	p<0.0001
log likelihood	-7.0605		-10.2134		-6.4416		-14.8992	
AIC	28.12099		30.42678		28.8832		39.79834	
BIC	42.89711		41.06245		46.02828		50.74661	

Standard errors are given in parentheses. Parameter estimates and standard errors are rounded to five decimal places.

For the *traditional* approach, which was based on an efficiency score calculated using a smaller set of inputs and outputs, the results of Tobit regression are shown in Table 15. Again, the set of determinants changed in 2015–2018 and only three variables were significant during the whole period—dummy for big hospitals (BIG), which had a negative relationship with inefficiency; and the share of ICU beds (ICU.SHARE) and the ratio of doctors to beds (DOC.BEDS), which both had a positive relationship with inefficiency. The ratio of nurses to beds (NURS.BEDS) was significant in 2015–2017 and had a negative relationship with inefficiency, leading to its decrease. The presence of the ED in the hospital (ED) was significant only in 2018, but—contrary to expectations—the parameter was negative. On the other hand, the ratio of the number of beds in ED to the total number of beds (ED.SHARE) was significant during the whole period except for 2017 and the parameter had a positive relationship with inefficiency. It may be concluded that the negative impact of ED and ICU on efficiency is confirmed.

**Table 15:** Results of Tobit regression of inefficiency on hospital characteristics (*traditional* approach).

Year Variable	2015		2016		2017		2018	
	Parameter	p-value	Parameter	p-value	Parameter	p-value	Parameter	p-value
	(std.error)		(std.error)		(std.error)		(std.error)	
const	0.1847 (0.0623)	0.003	0.2069 (0.0598)	0.0005	0.2284 (0.0589)	0.0001	-0.0432 (0.11)	0.6945
ED.SHARE	5.9717 (2.7645)	0.0308	5.6978 (2.5698)	0.0266			10.451 (4.0408)	0.0097
ICU.SHARE	5.1678 (2.2157)	0.0197	4.2426 (1.6638)	0.0108	7.0862 (2.237)	0.0015	4.816 (1.8876)	0.0107
DOC.BEDS	0.1231 (0.0492)	0.0124	0.153 (0.0517)	0.0031	0.2469 (0.0669)	0.0002	0.0822 (0.029)	0.0046
NURS.BEDS	-0.0449 (0.0186)	0.016	-0.0497 (0.0184)	0.0069	-0.0755 (0.0255)	0.0031		
BIG	-0.1964 (0.0645)	0.0023	-0.2329 (0.0705)	0.001	-0.3277 (0.0835)	0.0001	-0.1879 (0.0561)	0.0008
DOC.SHARE							1.6086 (0.6061)	0.008
ED							-0.2416 (0.1121)	0.0311
sima (SE)	0.241253	(0.02877)	0.243508	(0.02914)	0.273869	(0.03827)	0.240321	(0.02435)
chi2	30.68121	p<0.0001	31.88252	p<0.0001	27.89953	p<0.0001	36.0894	p<0.0001
log likelihood	-6,18015		-7,23628		-13,6537		-7,02787	
AIC	26,3603		28,47257		39,30735		30,05574	
BIC	41,36224		43,58475		52,26065		47,8118	

Standard errors are given in parentheses. Parameter estimates and standard errors are rounded to five decimal places.

## Discussion

The present paper extends the literature both on hospital efficiency and the determinants of inefficiency by proposing two new potential determinants: ED and ICU. To the best of the author’s knowledge, these two determinants had not been previously considered, particularly in the case of Polish county hospitals.

The hospitals included in the study come from all regions of Poland. The data used were relatively current and the analysis covered five years. Moreover, the study was based on hospitals as whole organizations, not individual departments. This allowed for the inclusion of some cost categories, for example, outsourcing services. It is also the first study of the Polish county hospitals to analyze the determinants in subgroups, controlling for the others.

The present study did not thoroughly confirm the findings presented by Lachowska [22], who reported higher efficiency in non-public healthcare providers in the West Pomeranian province. In the present study, mean and median efficiencies were higher for commercialized hospitals in almost all of the analyzed subgroups. However, in general, the differences in means turned out to be insignificant and the distribution of the numbers of efficient and inefficient hospitals was not significantly affected by ownership. Moreover, results from the Tobit models did not confirm the significant effect of the type of ownership, when controlling for other potential determinants. On the one hand, insignificant differences in efficiency between public and commercialized hospitals are surprising. On the other hand, it is important to mention that the situation in Polish hospitals is generally unfavorable.

Increasing wages in the economy and rising energy prices impact both public and commercialized units. By comparing the results obtained here to those reported by Lachowska [22], it may be hypothesized that over the years external conditions have led to a decrease of efficiency in both groups and the blurring of the differences between them. This leads to the assumption that even if public hospitals were less efficient than commercialized ones, their owners cannot afford to maintain this inefficiency over a long period of time, and thus the efficiency of hospitals is in a way forced by the environment. Unfortunately, the data used here did not allow the verification of this hypothesis. It is also worth noting that if highly efficient outliers existed, they usually appeared in the subgroups of commercialized hospitals.

The effect of the other two determinants, ED and ICU, is more clearly seen. Firstly, results from the *general* approach show that the presence of an ED in the hospital structure significantly lowered the efficiency in the whole group of hospitals and also had a significant effect on whether a hospital was considered efficient or not (2015–2017). Differences in mean efficiency were also statistically significant for ED, in the case of the *detailed* approach and the subgroup of hospitals with an ICU (whole period), as well as for the subgroup of public hospitals (3 years). In the case of the *traditional* approach, the effect of ED was not visible for the whole group. On the other hand, differences in mean efficiency were statistically significant in the cases of the subgroup of commercialized hospitals (3 years) and the subgroup of hospitals with an ICU. However, the impact of ICU on whether a hospital was considered efficient was significant both in the whole sample and in the *general* approach.

The negative impact of ICU and ED was confirmed in the Tobit models, in which variables representing the number of beds were significant. Based on the results of the *general* DEA approach, ED.SHARE was significant over the whole period and the parameters were positive. The dummy for ICU was significant only in 2015, but the sign of the parameter was in line with expectations. When Tobit model estimates based on the results of the *traditional* DEA approach were considered, both ICU.SHARE and ED.SHARE were significant over almost the whole period and the parameter had a positive relationship with inefficiency. The dummy for ED was significant only in 2018 when it had a negative relationship with inefficiency. Nonetheless, in the author’s opinion, both of these determinants were significant with an additional conclusion that not only the presence of such department/unit matters for efficiency but its relative size is important as well.

Results from the Tobit models bring some additional insight into other factors affecting efficiency, such as hospital size [5,10,11,13] or staff structure [10,13]. The results presented here suggest that the size of the hospital plays an important role. Results from the *traditional* Tobit model showed that bigger hospitals were less inefficient which may be surprising considering the complexity of such a structure. On the other hand, the results showed that bigger hospitals were less inefficient. Fixed costs may lead to lower inefficiency in their case. Moreover, bigger hospitals may have a stronger position in the market, for example being able to negotiate more favorable prices for products and services. If necessary, they can also afford to use staff and resources more flexibly. It is worth mentioning that Cheng et al. [13] concluded that the biggest hospitals were negatively related to efficiency. Results from the *general* Tobit model presented in the current paper confirmed the conclusion from Chowdhury and Zelenyuk [11] about the higher efficiency of smaller hospitals. The higher efficiency in these cases may origin from the fact that they are usually less complex units, and thus easier to manage. It should be noted, however, that in all these studies, size categories were defined differently.

The impact of the nurses-to-beds ratio on inefficiency was inconclusive (positive in the *general* model and negative in the *traditional* one) while in the literature the effect is negative. Cheng et al. [13] stated that the relationship between the ratio of beds to nurses and efficiency was positive. The impact of the doctors-to-beds ratio on inefficiency is inconclusive as well (positive in the *traditional* model and negative or positive in the *general* one). In comparison with Ali et al. [10], the doctor-to-total-staff ratio was reported to be negatively related to the inefficiency of hospitals. In the present study, this variable turned out to have a positive relationship with inefficiency. Nurses share in total staff was significant only in one case and had a negative relationship with inefficiency.

The study has several limitations. Firstly, it was not possible to use case-mix data, which are among the most often used determinants of efficiency, as such information was not available in the dataset. Secondly, there may be a relationship between the efficiency and location of the hospital [9,11]. The present study did not include information about the region in which the hospital is located or its socio-economic characteristics which may have influenced, for example, the number of patients or most treated conditions. The reason for this decision was the very limited number of hospitals from some of the 16 Polish first-level administrative regions (i.e. voivodeships). Due to this, the author decided not to include the geographical aspect in the analysis as the result might have been biased. One drawback is related to the method itself. DEA results are deterministic, strictly dependent on the sample, which means that the efficiency scores are prone to change if a new hospital is included in the study. The last limitation is connected to the small number of hospitals of certain types, which did not allow conclusions to be drawn regarding some of the determinants in the subgroups.

## Conclusions

The topic addressed in the present paper is especially significant in view of the changes that have taken place in the Polish healthcare sector since 1997 [2,20]. The COVID-19 pandemic demonstrated a detailed and profound view of the problems in Polish hospitals, as well as in the whole sector. While the aim of the paper was not to discuss the difficulties that were encountered by the healthcare facilities in 2020, the analysis presented may prove useful in determining whether signs of the problems were visible before the pandemic. Possibilities of relative comparisons constitute a second important output. It is worth mentioning that this is the first analysis of this type conducted on such a group of Polish county hospitals from different regions of Poland and of different complexity.

The hypotheses posed in the introduction were only partially confirmed. It was shown that both ED and ICU have an impact on efficiency. However, the lack of effect of the type of ownership on efficiency is surprising, but may be attributed to the overall unfavorable situation of Polish county hospitals. On the other hand, the author was able to provide additional insight into the determinants of the inefficiency of this groups of hospitals—the impact of hospital size and several ratios connected to staff are significant.

It is difficult to forecast how the situation of the Polish healthcare system will be after the COVID-19 pandemic is over. However, it seems appropriate that decision makers should take into account the relationships between hospital characteristics and inefficiency concluded in the present analysis.

## References

[1] Nojszewska E, Sielska A, Gołąb-Bełtowicz D. Raport z badania sytuacji finansowej szpitali powiatowych–Szklarska Poręba’19. [Internet] 2019 [Cited: 2020 Nov 30] Available from: http://ozpsp.pl/slider/raport-na-temat-sytuacji-szpitali-powiatowych/.

[2] Nojszewska E, Malinowski W, Sikorski S. Komercyjne świadczenie usług medycznych przez szpitale publiczne. Warszawa: Wolter Kluwer Polska; 2017.

[3] de Souza AA, Moreira DR, Avelar EA, de Faria Marques AM, Lucchesi Lara A. Data envelopment analysis of efficiency in hospital Organisations. Int J Business Innovation and Research. 2014; 8(3): 316–332.

[4] Kalhor R, Amini S, Sokhanvar M, Lotfi F, Sharifi M, Kakemam E. Factors affecting the technical efficiency of general hospitals in Iran: data envelopment analysis. J Egypt Public Health Assoc. 2016 Mar;91(1):20–5. doi: 10.1097/01.EPX.0000480717.13696.3c . 27110856

[5] Mujasi PN, Asbu EZ, Puig-Junoy J. How efficient are referral hospitals in Uganda? A data envelopment analysis and tobit regression approach. BMC Health Serv Res. 2016; 16:230. doi: 10.1186/s12913-016-1472-9 27391312 PMC4939054

[6] Staat M. Efficiency of hospitals in Germany: a DEA-bootstrap approach. Appl Econ. 2006; 38(19): 2255–2263, doi: 10.1080/00036840500427502

[7] Tiemann O, Schreyögg J. Effects of Ownership on Hospital Efficiency in Germany. BuR—Business Research, VHB–Verband der Hochschullehrer für Betriebswirtschaft, German Academic Association of Business Research, Göttingen. 2009; 2(2): 115–145. doi: 10.1007/BF03342707

[8] Valdmanis VG, Rosko MD, Mutter RL. Hospital Quality, Efficiency, and Input Slack Differentials. HSR: Health Services Research. 2008; 43:5, Part II (October 2008): 1830–1848. 18783457 10.1111/j.1475-6773.2008.00893.x PMC2654161

[9] Campanella P, Azzolini E, Izzi A, Pelone F, De Meo C, La Milia Specchia ML, et al. Hospital efficiency: how to spend less maintaining quality? Annali dell’Istituto Superiore Di Sanita. 2017; 53 (1): 46–53. doi: 10.4415/ANN_17_01_10 28361805

[10] Ali M, Debela M, Bamud T. Technical efficiency of selected hospitals in Eastern Ethiopia. Health Econ Rev. 2017; 7(24): 1–13. doi: 10.1186/s13561-017-0161-7 28634925 PMC5478555

[11] Chowdhury H, Zelenyuk V. Performance of hospital services in Ontario: DEA with truncated regression approach. Omega. 2016; 63: 111–122. doi: 10.1016/j.omega.2015.10.007

[12] Torabipour A, Najarzadeh M, Arab M, Farzianpour F, Ghasemzadeh R (2014) Hospitals Productivity Measurement Using Data Envelopment Analysis Technique, Iranian J Publ Health. 2014; 43(11): 1576–1581. 26060727 PMC4449509

[13] Cheng Z, Tao H, Cai M, Lin H, Lin X, Shu Q, et al. Technical efficiency and productivity of Chinese poviat hospitals: an exploratory study in Henan province, China. BMJ Open. 2015; 5:e007267. doi: 10.1136/bmjopen-2014-007267 26353864 PMC4567660

[14] Kirigia JM, Asbu EZ. Technical and scale efficiency of public community hospitals in Eritrea: an exploratory study. Health Econ Rev. 2013; 3:6. doi: 10.1186/2191-1991-3-6 23497525 PMC3605339

[15] Sielska A, Nojszewska E. Efficiency of Polish county hospitals. In: Papież M, Śmiech S, editors. The 14th Professor Aleksander Zelias International Conference on Modelling and Forecasting of Socio-Economic Phenomena. Conference Proceedings. Krakow: Wydawnictwo Małopolskiej Szkoły Administracji Publicznej Uniwersytetu Ekonomicznego w Krakowie. p. 145–154. Available from: http://pliki.konferencjazakopianska.pl/proceedings_2020/pdf/2020_Monografia-15.pdf.

[16] Cygańska M, Kludacz-Alessandri M, Syrrakos D. Measuring Efficiency in Clinical Departments Using the DEA Approach—A Case of the Polish Hospital. In: Nesleha J, Hampl F, Svoboda M, editors. European Financial Systems 2018: Proceedings Of The 15th International Scientific Conference; 2018 Jun 25–26; Brno, Czech Republic. 2018, p. 61–69.

[17] Grzesiak S, Wyrozębska A. Wykorzystanie Metody Dea (Analizy Obwiedni Danych) do Oceny Efektywności Technicznej Oddziałów Szpitalnych. Studia i Prace Wydziału Nauk Ekonomicznych i Zarządzania Uniwersytetu Szczecińskiego. 2014; 36(2): 253–272.

[18] Miszczyńska K. Improving managerial decisions in the health care sector. Application of PROMETHEE II method to public hospitals. Operations Research and Decisions. 2021; 4: 65–79.

[19] Łagowski P. Ocena efektywności świadczenia usług medycznych przez jednostki szpitalne z uwzględnieniem formy organizacyjno-prawnej podmiotu. Studia i Prace Kolegium Zarządzania i Finansów. 2018; 167:95–110.

[20] Ćwiąkała-Małys A, Durbajło-Mrowiec M, Łagowski P. Diagnostyka efektywności wykorzystania zasobów lecznictwa szpitalnego. [Internet]. Wrocław: E-Wydawnictwo. Prawnicza i Ekonomiczna Biblioteka Cyfrowa. Wydział Prawa, Administracji i Ekonomii Uniwersytetu Wrocławskiego; 2020 [cited 2020 Nov 30]. Available from: https://www.bibliotekacyfrowa.pl/publication/116241. doi: 10.34616/23.20.005

[21] Rój J. Efektywność usługowa jako kryterium wyboru mechanizmu finansowania szpitali. Ruch Prawniczy, Ekonomiczny i Socjologiczny. 2003; 65(4): 153–171.

[22] Lachowska A. Efficiency of public and nonpublic primary health care providers in Poland. Engineering Management in Production and Services. 2017; 9(2): 57–63.

[23] Jewczak M, Żółtaczek A. Ocena efektywności technicznej podmiotów sektora opieki zdrowotnej w Polsce w latach 1999–2009 w ujęciu przestrzenno-czasowym na przykładzie szpitali ogólnych. Problemy Zarządzania. 2011; 9(3): 194–210.

[24] Kocisova K, Hass-Symotiuk M, Kludacz-Alessandri M. Use of the DEA Method to Verify the Performance Model for Hospitals. Business Administration and Management. 2018; 4(XXI): 125–140. doi: 10.15240/tul/001/2018-4-009

[25] Kujawska J. Wykorzystanie metod nieparametrycznych do pomiaru efektywności opieki szpitalnej w Polsce. Studia Ekonomiczne. 2013; 168:111–124.

[26] Luptáčik M. Scarcity and Efficiency. In: Mathematical Optimization and Economic Analysis. In: Luptáčik M. Springer Optimization and Its Applications, vol 36. New York: Springer; 2010. p. 3–24. doi: 10.1007/978-0-387-89552-9_1

[27] Azreena E, Muhamad Hanafiah Juni, Rosliza AM. A Systematic Review of Hospital Inputs and Outputs in Measuring Technical Efficiency Using Data Envelopment Analysis. International Journal of Public Health and Clinical Sciences. 2018; 5(1): 17–35.

[28] Ravaghi H, Afshari M, Isfahani P, Bélorgeot VD. A systematic review on hospital inefficiency in the Eastern Mediterranean Region: sources and solutions. BMC Health Serv Res. 2019; 19(830). doi: 10.1186/s12913-019-4701-1 PMC6852759 31718648

[29] Zakowska I, Godycki-Cwirko M. Data envelopment analysis applications in primary health care: a systematic review. Family Practice. 2020; 147–153. doi: 10.1093/fampra/cmz057 31605609

[30] Almeida A, Cima J. Demand uncertainty and hospital costs: An application to Portuguese public hospitals. European J Health Econ. 2015; 16(1): 35–45. doi: 10.1007/s10198-013-0547-3 24310509

[31] Hyman J, Folland S. Uncertainty and Hospital Costs. South Econ J [Internet]. Oct. 1972 [cited 2020 Dec 22]; 39(2): 267–273.

[32] Ludwig M, Van Merode F, Groot W. Principal agent relationships and the efficiency of hospitals. The European journal of health economics: HEPAC: health economics in prevention and care. 2010; 11(3): 291–304. doi: 10.1007/s10198-009-0176-z 19655184 PMC2860099

[33] Kornai J. The Soft Budget Constraint. Kyklos. 1986; 39: 3–30. doi: 10.1111/j.1467-6435.1986.tb01252.x 19377869

[34] Vahabi M. The Soft Budget Constraint: A Theoretical Clarification. [Internet] Recherches Économiques De Louvain/Louvain Economic Review. 2001 [Cited: 2020 Dec 8]; 67(2): 157–195. Available from http://www.jstor.org/stable/40724317.

[35] Wright DJ. Soft Budget Constraints in Public Hospitals. Health Economics. 2016; 25: 578–590, Published online 5 March 2015 in Wiley Online Library (wileyonlinelibrary.com). doi: 10.1002/hec.3174 25740723

[36] Aigner D, Lovell C, Schmidt P. Formulation and estimation of stochastic frontier production function models. Journal of Econometrics. 1977; 6(1): 21–37.

[37] Meeusen W, van Den Broeck J. Efficiency estimation from Cobb–Douglas production functions with composed error. International Economic Review. 1977; 18(2): 435–444.

[38] Andersen P, Petersen NC. A procedure for ranking efficient units in data envelopment analysis. Manage Sci. 1993; 39:1261–1264. doi: 10.1287/mnsc.39.10.1261

[39] Zhu J. Quantitative Models for Performance Evaluation and Benchmarking. Data Envelopment Analysis with Spreadsheets and DEA Excel Solver. Springer Science+Business Media New York; 2003. doi: 10.1007/978-1-4757-4246-6

[40] R Core Team. R: A language and environment for statistical computing. [Software] R Foundation for Statistical Computing, Vienna, Austria. 2020 [cited: 2020 Nov 10—Dec 08] Available from: https://www.R-project.org/.

[41] Coll-Serrano V, Bolos V, Benitez Suarez R. deaR: Conventional and Fuzzy Data Envelopment Analysis. R package version 1.2.1. [Software] 2020 [cited: 2020 Nov 10—Dec 08] Available from: https://CRAN.R-project.org/package=deaR.

[42] Golany B, Roll Y. An Application Procedure for DEA. Omega. 1989; 17: 237–250. doi: 10.1016/0305-0483(89)90029-7

[43] Li Q, Tian L, Jing X, Chen X, Li J, Chen H. Efficiency and scale effect of poviat public hospitals in Shandong Province, China: a cross-sectional study. BMJ Open. 2020; 10:e035703. doi: 10.1136/bmjopen-2019-035703 32540890 PMC7299019

[44] Park S. Analyzing the efficiency of small and medium-sized enterprises of a national technology innovation research and development program, SpringerPlus 2014, 3:403, doi: 10.1186/2193-1801-3-403 25120949 PMC4128955

[45] Selker H, Beshansky J, Pauker S, Kassirer J. The Epidemiology of Delays in a Teaching Hospital: The Development and Use of a Tool That Detects Unnecessary Hospital Days. [Internet] Medical Care. 1989 [Cited: 2020 Dec 22]; 27(2), 112–129. Available from: www.jstor.org/stable/3765136. doi: 10.1097/00005650-198902000-00003 2918764

[46] Storrow AB, Zhou C, Gaddis G, Han JH, Miller K, Klubert D, et al. Decreasing Lab Turnaround Time Improves Emergency Department Throughput and Decreases Emergency Medical Services Diversion: A Simulation Model. Academic Emergency Medicine. Official Journal of the Society of Academic Emergency Medicine. Special Issue: Proceedings of The 2008 AEM Consensus Conference: The Science of Simulation in Healthcare: Defining and Developing Clinical Expertise 2008; 15(11S): 1130–1135. 10.1111/j.1553-2712.2008.00181.x 18638034

[47] Cottrell A, Lucchetti R. Gnu Regression, Econometrics and Time-series Library. Version 2018C [Software] [cited: 2020 Nov 10—Dec 08] Available from: http://gretl.sourceforge.net

