# Correction: Determinants of hospital inefficiency. The case of Polish county hospitals

**DOI:** 10.1371/journal.pone.0260461

**Published:** 2021-11-18

**Authors:** The PLOS ONE Staff

There is an error in the author’s name; the publisher apologizes for the error. The correct name is: Agata Sielska. The correct citation is: Sielska A (2021) Determinants of hospital inefficiency. The case of Polish county hospitals. PLoS ONE 16(8): e0256267. https://doi.org/10.1371/journal.pone.0256267

## References

[1] Sielskas A (2021) Determinants of hospital inefficiency. The case of Polish county hospitals. PLoS ONE 16(8): e0256267. 10.1371/journal.pone.0256267 34403449 PMC8660014

